# Supplementary material for: RNF219 attenuates global mRNA decay through inhibition of CCR4-NOT complex-mediated deadenylation
Source: Nat Commun. 2021 Dec 9;12:7175. doi: 10.1038/s41467-021-27471-6 (PMC8660800; doi:10.1038/s41467-021-27471-6)
Supplement: Supplementary file 1 — Supplementary Information [file 41467_2021_27471_MOESM1_ESM.pdf]

Supplementary Information

RNF219 attenuates global mRNA decay through inhibition of CCR4-NOT complex-mediated deadenylation

Fabian Poetz, Joshua Corbo, Yevgen Levdansky, Alexander Spiegelhalter, Doris Lindner, Vera Magg, Svetlana Lebedeva, Jörg Schweiggert, Johanna Schott, Eugene Valkov, and Georg Stoecklin

Supplementary Figures

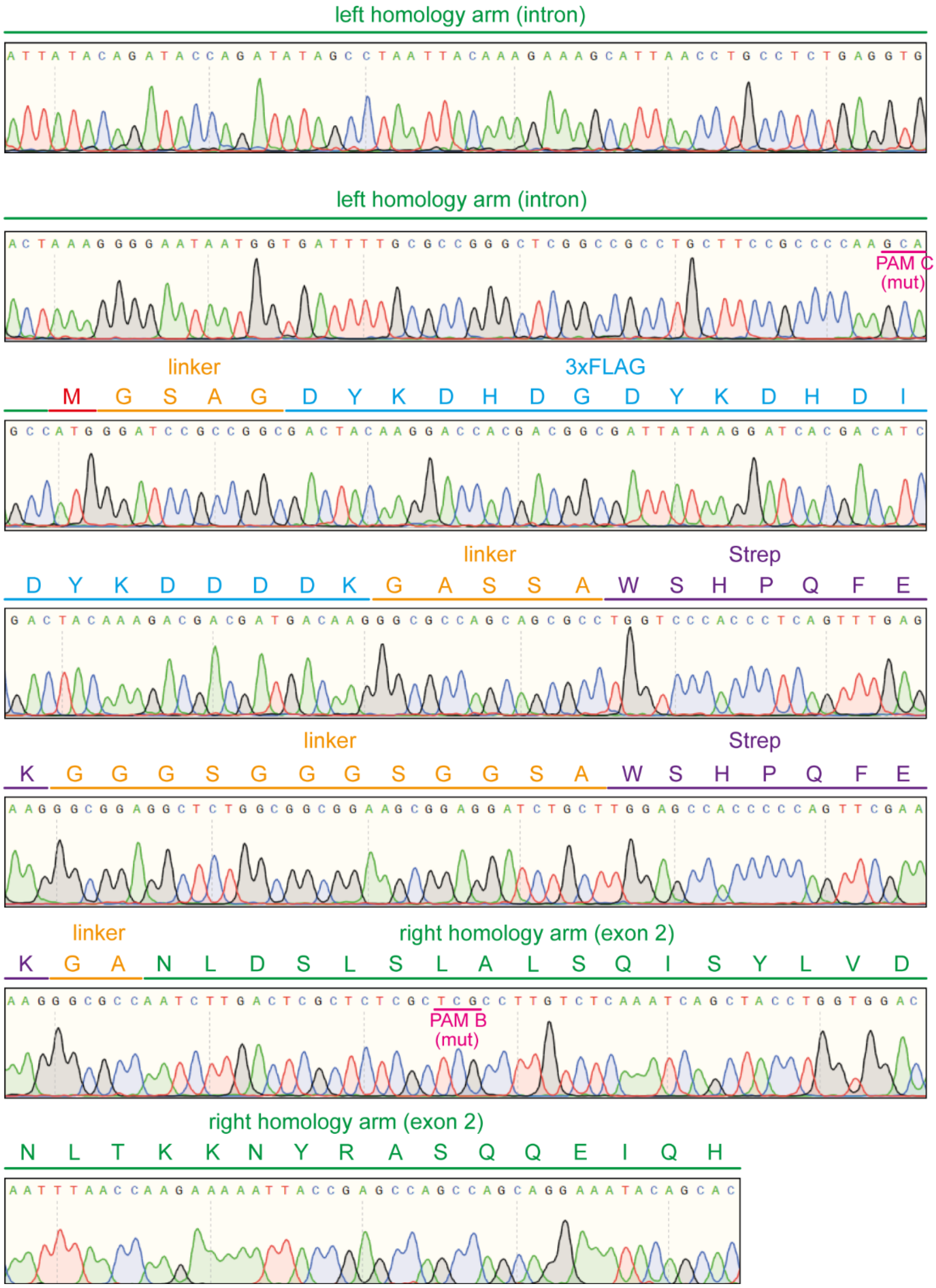

Supplementary Figure 1 | Verification of CRISPR/Cas9-mediated genome editing of *CNOT1* following homology-directed repair (related to Figure 1). Sequencing chromatogram of the edited *CNOT1* genomic locus containing the desired 3xFLAG-2xStrep

insertion downstream of the ATG start codon in clone #47. A region of the *CNOT1* genomic locus spanning the edited site was PCR amplified, sub-cloned and subjected to Sanger sequencing. The amino acid sequence is indicated above the nucleotide sequence and color-coded; the ATG start codon is highlighted in red; mutated PAMs are highlighted in purple.

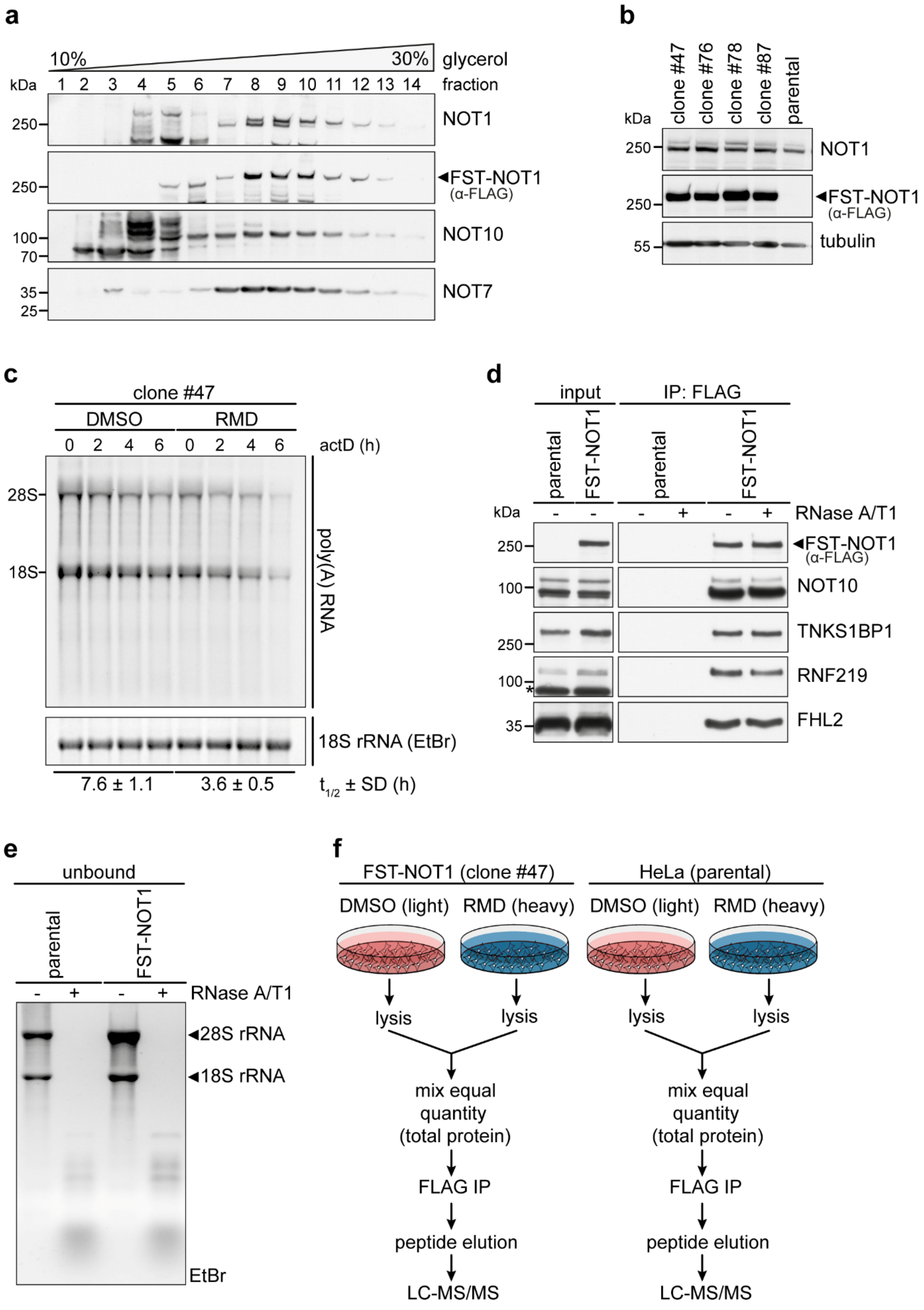

a
10%
30%
glycerol
fraction
kDa
1 2 3 4 5 6 7 8 9 10 11 12 13 14
250
NOT1
FST-NOT1
(α-FLAG)
NOT10
100
70
NOT7
35
25
b
clone #47
clone #76
clone #78
clone #87
parental
NOT1
tubulin
55
c
clone #47
DMSO
RMD
0 2 4 6 0 2 4 6
actD (h)
28S
18S
poly(A) RNA
18S rRNA (EtBr)
7.6 ± 1.1
3.6 ± 0.5
t1/2 ± SD (h)
d
input
IP: FLAG
parental
FST-NOT1
RNase A/T1
TNKS1BP1
RNF219
FHL2
e
unbound
28S rRNA
18S rRNA
EtBr
f
FST-NOT1 (clone #47)
HeLa (parental)
DMSO (light)
RMD (heavy)
lysis
mix equal quantity (total protein)
FLAG IP
peptide elution
LC-MS/MS

Supplementary Figure 2 | Characterization of HeLa-FST-NOT1 clone #47 and purification of the endogenous CCR4-NOT complex (related to Figure 1). (**a**) Co-sedimentation analysis of CCR4-NOT subunits by 10-30% linear glycerol gradient fractionation and western blotting from HeLa-FST-NOT1 clone #47. The core CCR4-NOT complex elutes in fraction 7-14. (**b**) Western blot analysis of different HeLa-FST-NOT1 clones next to parental HeLa cells using antibodies as indicated. (**c**) Measurement of bulk poly(A) RNA stability in HeLa-FST-NOT1 clone #47 after treatment with 20 nM RMD or an equal volume of solvent (DMSO) for 16 hours. Transcription was shut-off by addition of 5 µg/ml actinomycin D (actD) prior to total RNA extraction at regular time intervals. Poly(A) RNA was visualized by northern blot analysis using an oligo $d(T)_{18}$ probe, and 18S rRNA was visualized by ethidium bromide (EtBr) staining after blotting. The poly(A) RNA signal normalized to 18S rRNA was used for calculating its half-life; values are presented as mean ± SD (n = 3). (**d**) The CCR4-NOT complex was purified from HeLa-FST-NOT1 or parental HeLa cells by FLAG IP in the absence or presence of RNase A/T1. Proteins were detected by western blot analysis using antibodies as indicated; the asterisk denotes a non-specific band. (**e**) Unbound material from (d) was subjected to total RNA extraction followed by denaturing formaldehyde-containing 1.1% agarose gel electrophoresis. RNA was stained with EtBr. (**f**) Scheme illustrating the SILAC-based approach to identify changes in the composition of the CCR4-NOT complex following treatment with the class I-specific HDAC inhibitor Romidepsin (RMD). Source data for panels **a-e** are provided as a Source Data file.

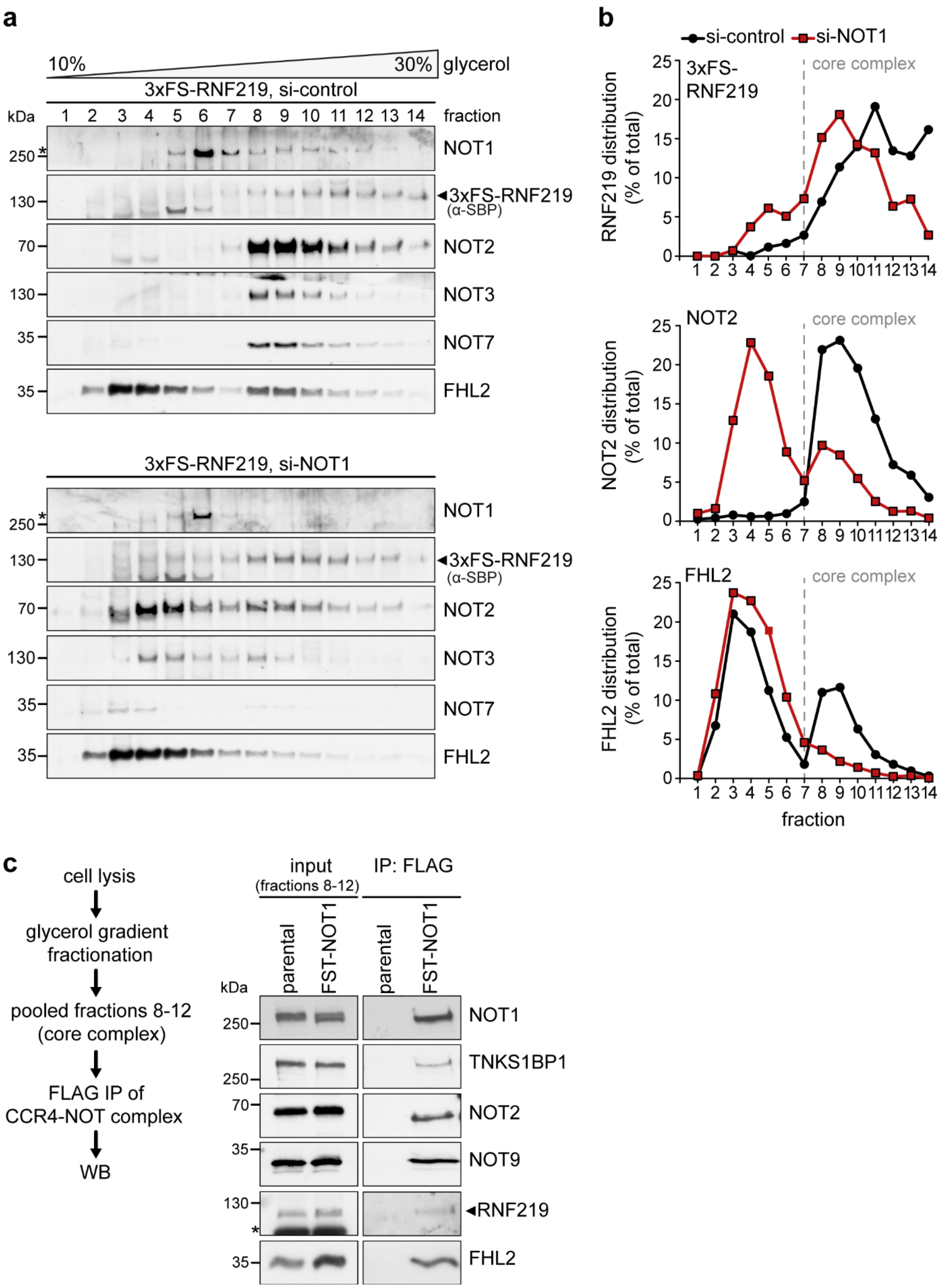
a
10%
30%
glycerol
3xFS-RNF219, si-control
kDa
1 2 3 4 5 6 7 8 9 10 11 12 13 14
fraction
250
*
NOT1
130
◂3xFS-RNF219
(α-SBP)
70
NOT2
130
NOT3
35
NOT7
35
FHL2
3xFS-RNF219, si-NOT1
250
*
NOT1
130
◂3xFS-RNF219
(α-SBP)
70
NOT2
130
NOT3
35
NOT7
35
FHL2
b
si-control
si-NOT1
3xFS-
RNF219
core complex
RNF219 distribution
(% of total)
NOT2
core complex
NOT2 distribution
(% of total)
FHL2
core complex
FHL2 distribution
(% of total)
0 5 10 15 20 25
1 2 3 4 5 6 7 8 9 10 11 12 13 14
fraction
c
cell lysis
glycerol gradient
fractionation
pooled fractions 8-12
(core complex)
FLAG IP of
CCR4-NOT complex
WB
input
(fractions 8-12)
IP: FLAG
parental
FST-NOT1
parental
FST-NOT1
kDa
250
NOT1
250
TNKS1BP1
70
NOT2
35
NOT9
130
*
◂RNF219
35
FHL2

Supplementary Figure 3 | RNF219 and FHL2 stably interact with the CCR4-NOT complex (related to Figure 1). (**a**) Co-sedimentation analysis of CCR4-NOT subunits by 10-30% linear glycerol gradient fractionation and western blotting. HeLa cells stably expressing 3xFS-RNF219 were transfected twice with either control or NOT1-targeting siRNAs over a period of 72 hours before preparation of cell lysates and ultracentrifugation. The core CCR4-NOT complex elutes in fractions 7-14; the asterisks denote non-specific bands. (**b**) Distribution of 3xFS-RNF219, NOT2 and FHL2 across the glycerol gradient was quantified from blots shown in (a); n = 1. (**c**) Scheme illustrating the experimental workflow to assess association of RNF219 and FHL2 with the CCR4-NOT complex (left panel). Following separation of lysate from parental or FST-NOT1 HeLa cells on a 10-30% linear glycerol gradient by ultracentrifugation and fractionation, the CCR4-NOT complex was purified from pooled fractions 8-12 by FLAG IP. Co-precipitating proteins were analyzed by western blotting (right panel). Source data for panels **a-c** are provided as a Source Data file.

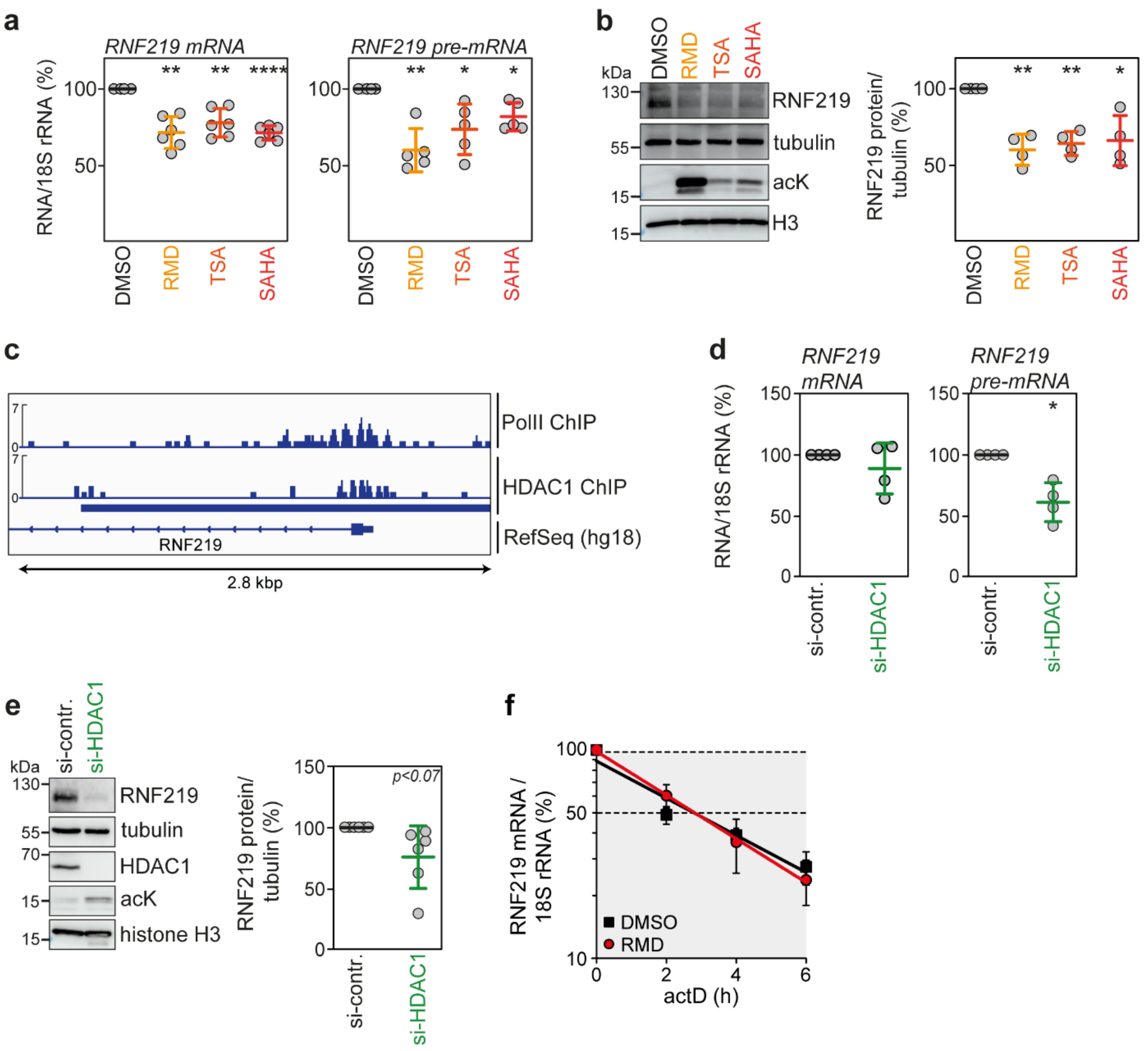

Supplementary Figure 4 | HDAC inhibition downregulates RNF219 expression (related to Figure 1). (**a**) *RNF219* mRNA and pre-mRNA levels were examined by qRT-PCR following treatment of HeLa cells with 20 nM RMD, 500 nM Trichostatin A (TSA), 500 nM suberoylanilide hydroxamic acid (SAHA) or an equal volume of solvent (DMSO) for 16 hours. 18S rRNA was used for normalization; values are presented as mean ± SD. p-values were calculated using a two-sided, one-sample t-test (*RNF219* mRNA, n = 6, RMD $p = 0.0011$, TSA $p = 0.0021$, SAHA $p < 0.0001$; *RNF219* pre-mRNA, n = 5, RMD $p = 0.0032$, TSA $p = 0.0236$, SAHA $p = 0.0117$). (**b**) HeLa cells were treated as described in (a) and RNF219 protein levels were examined by western blot analysis (left side). RNF219 protein expression is quantified on the right side using tubulin for normalization; values are presented as mean ± SD (n = 4). p-

values were calculated using a two-sided, one-sample t-test; RMD $p = 0.0042$, TSA $p = 0.0028$, SAHA $p = 0.0258$. (**c**) Integrative Genomics Viewer tracks of RNA polymerase II (PolII) and HDAC1 ChIP-Seq data derived from $CD4^+$ T-cells[1]. (**d**) *RNF219* mRNA and pre-mRNA levels were examined by qRT-PCR following transfection of HeLa cells with either control or HDAC1-targeting siRNAs for 72 hours. 18S rRNA was used for normalization; values are presented as mean ± SD (n = 4). *p*-value was calculated using a two-sided, one-sample t-test; $p = 0.0165$. (**e**) HeLa cells were transfected with siRNAs as described in (d) and RNF219 protein levels were examined by western blot analysis (left side). RNF219 protein expression is quantified on the right side using tubulin for normalization; values are presented as mean ± SD (n = 6). *p*-value was calculated using a two-sided, one-sample t-test; $p = 0.0671$. (**f**) Decay of *RNF219* mRNA was measured in HeLa cells following treatment with 20 nM RMD or an equal volume of solvent (DMSO) for 16 hours. Transcription was shut-off with 5 µg/ml actinomycin D (actD). Total RNA was extracted at regular time intervals and analyzed by qRT-PCR. 18S rRNA was used for normalization; values are presented as mean ± SEM (n = 3). * $p < 0.05$, ** $p < 0.01$, **** $p < 0.0001$. Source data for panels **a-b** and **d-f** are provided as a Source Data file.

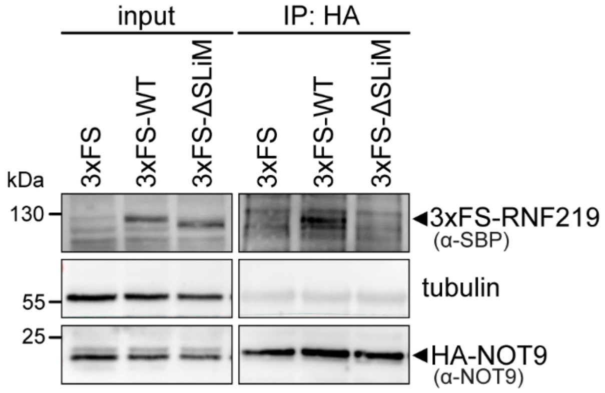

Supplementary Figure 5 | Co-precipitation of RNF219 with HA-NOT9 requires its C-terminal SLiM (related to Figure 2). HA-NOT9 was transiently transfected into HeLa cells stably expressing 3xFS-RNF219-WT or 3xFS-RNF219-ΔSLiM. Following HA IP, precipitating proteins were detected by western blot analysis using antibodies as indicated. Source data for this panel are provided as a Source Data file.

a

```
RNF219                                         RING
H. sapiens       MAQTVQNVTLSLTLPITCHICLGKVRQPVICINNHVFCSICIDLWLKNNSQCPACRVPIT 60
M. musculus      MAQTVQNVTLSLTLPITCHICLGKVRQPVVCTNNHVFCSICIDLWLKNNSQCPACRVPIT 60
G. gallus        MAQHGPSVTLSLTLPITCHICLGKVRHPVICVNNHVFCSICIEVWLKNNNQCPACRIPIT 60
X. laevis        MAQTVQNVTLSLTLPITCHICLGKVRQPVICVNHHVFCTVCIELWLKNNNQCPVCRVPIT 60
D. rerio         MAQSFQNVTLSLTLPISCQICLGKVRQPVICCNNHVFCSSCIKVWLEKSSQCPTCRVAIT 60
                 ***..::*********:*:*******:**:* *:****:.** .**:::.***.**::**
```

b

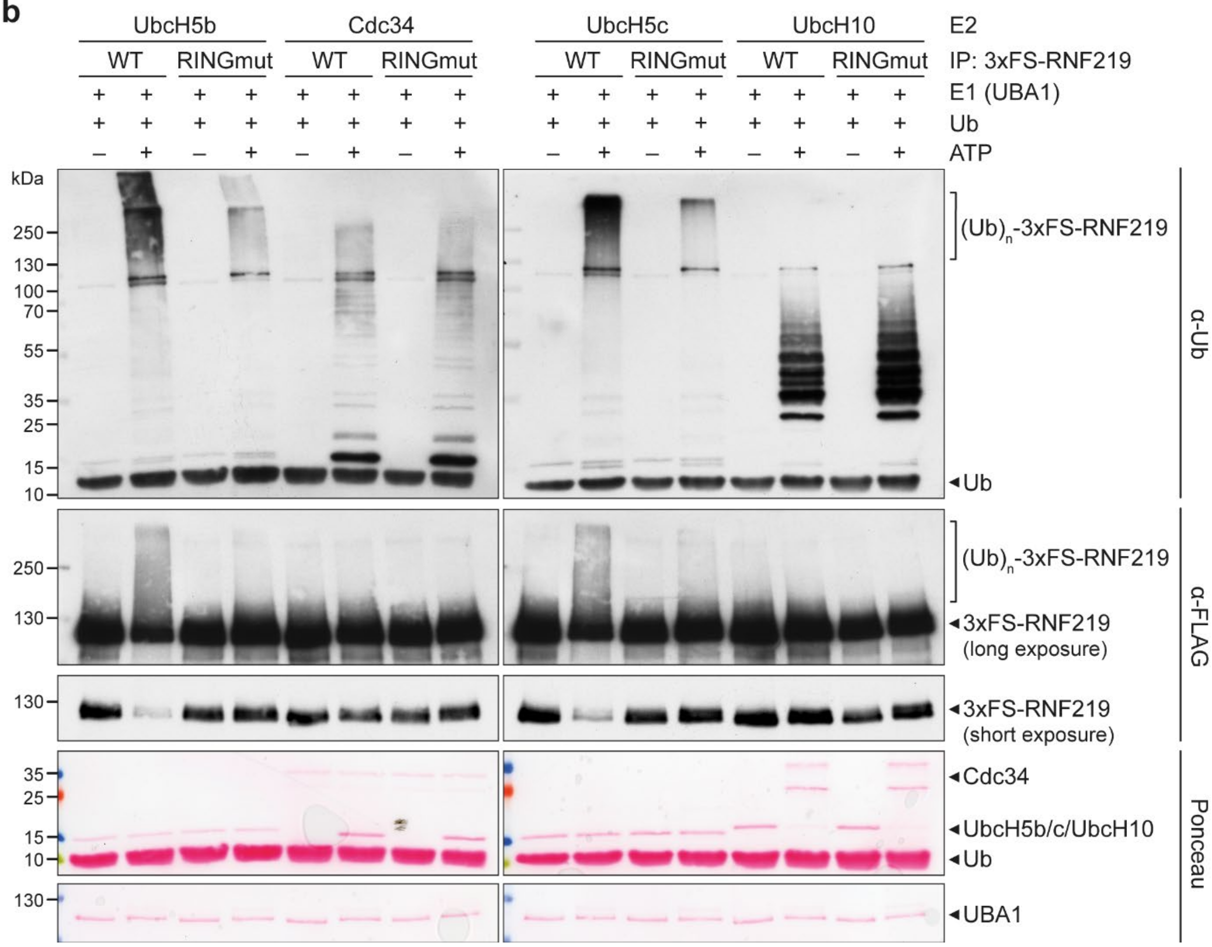

Supplementary Figure 6 | Conservation of the RNF219 RING domain and autoubiquitination of RNF219 (related to Figure 3). (**a**) Amino acid sequence conservation analysis of the N-terminal RING domain (green) of RNF219. Conservation was assessed between homologues in human (*Homo sapiens*), mouse (*Mus musculus*), chicken (*Gallus gallus*), frog (*Xenopus laevis*) and fish (*Danio rerio*). (**b**) *In vitro* ubiquitination assay showing autoubiquitination of 3xFS-RNF219-WT. 3x-FS-RNF219-WT or -RINGmut were affinity purified from transiently transfected HEK293 cells. Following 3xFLAG peptide elution,

RNF219 was incubated with recombinant E1 enzyme (UBA1), different E2 enzymes and ubiquitin in the absence or presence of 5 mM ATP. Reaction products were analyzed by western blotting using antibodies as indicated. Source data for panel **b** are provided as a Source Data file.

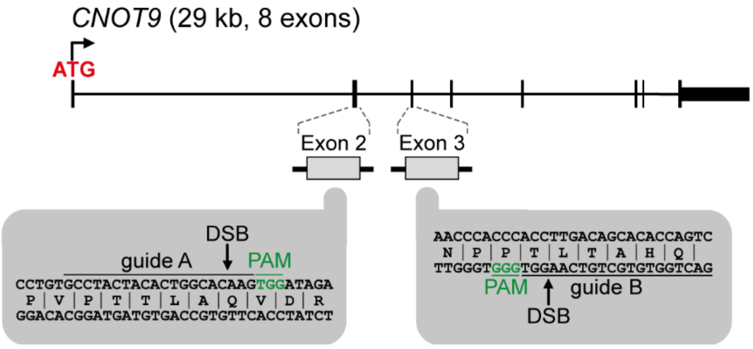

Supplementary Figure 7 | CRISPR/Cas9-mediated knock-out of NOT9 (related to Figure 3). Schematic illustration of the CRISPR/Cas9 strategy by which HeLa NOT9 KO cells were generated; DSB, double-strand break; PAM, protospacer adjacent motif.

a

RNF219-WT, NOT9 KO #5, allele 1

```
                                guide A     PAM A
parental       AAGCCTGTGCCTACTACACTGGCACAAGTGGATAGAGAAAAGATCTATCAGTGGATCAAT
NOT9 KO #5(WT) AAGCCTGTGCCTACTACACTGGCACAAGGTGG----------------------------
               ****************************  *

parental       GAGCTGTCCAGTCCTGAGACTAGGGAAAATGCTTTGCTGGAGCTAAGTAAGAAGCGAGAA
NOT9 KO #5(WT) ------------------------------------------------------------

parental       TCTGTTCCTGACCTTGCACCCATGCTGTGGCATTCATTTGGTACTATTGCAGCACTTTTA
NOT9 KO #5(WT) ------------------------------------------------------------

parental       CAGGTGGGTTCATGTCCATGATTGGCAGTTCAGTTCTTTTCATTACACTTGTATATTTCT
NOT9 KO #5(WT) ------------------------------------------------------------

parental       TTACCTTTGCCCAAATGATTTGAAGACTTTAGGAGGATGATTCTTTAAAAAACTGCATTT
NOT9 KO #5(WT) ------------------------------------GTGGGTTGATAGATGGATAAATAT
                                                     **..*...**.*....*..**.*

parental       GTAAGAATCTGTTTTAGAAAGGTCTGAGGTATTTGTTAAAATGCAGATTCTTGGATCCTT
NOT9 KO #5(WT) TTACA-ATTTCCTAAAAAATGGAAATCAGCATCA-------------------GATGAAT
                **.. **.*..*..*.**.**......*.**..                   ***...*

parental       ACCCAAGAGATTCAATTTAGTCAGTCTGAGGTGGGCCCTATAATCTATATTTTAATAAGC
NOT9 KO #5(WT) AAGGAATAACTAAAAATTAATTCCCATGGTAATAAAGC-------------AAAAAAAAA
               *...**.*..*..**.***.*.....**.........*             ..**.**..

parental       ACTTTCACCTCCCCCAGGTGATTGTGATGCAAGTGATCTAAGAACTACGCTTTGACGAAC
NOT9 KO #5(WT) AAAGGCAAATAAAATAGCTCTTAATTCTGTGCATCTTTTAAGAACTCAGTCATCAAAGAG
               *....**..*.....**.*..*..*..**....*..*.********..*...*.*...*.

parental       ACCGCTTTAGGAGTA--------CT-GTAAGATAAATCTACATGACAGGGGGAGGAAGGT
NOT9 KO #5(WT) GGAGCAGAAATAATGCTCATATCCTTTCATCACAAACTTACTTGGCTG----AGACTAGC
               ...**...*..*.*.         **...*..*.***..***.**.*.*    **....*.

parental       GGAAAGTAGAGTATAAGGACCACAAGGTACAATATATTCTATGGAAAAGAAATGGGTTTT
NOT9 KO #5(WT) TAAGACATTTCTACATTTGTCTCTAGGAAGATTTTTTTTTTTGAGACTCA----CATTTC
               ..*.*......**.*.....*.*.***.*.*.*.*.**.*.**..*...*    ...**.

parental       TGGGTTTTTTTGTTTGTTTTTCTTTTTTTTTTTTTTTGAGACGGAATCTCGCTCTGTCGC
NOT9 KO #5(WT) -----TACATTTGTCTCTAGGAAGATTTTTTTTTTTTGAGACGGAGTCTCGCTCTGTTAC
                    *...**..*...*......*********************.***********..*

parental       CCAGACTAGAGTGCAATGGTGCAATCCCGGCTCACTGCAACCTCTGCCTCCCGGGTTCAA
NOT9 KO #5(WT) CTGGGCTGGAGTGCAGTGGCACGATCTTGACTCACTGCAAGCTCTGCCTCCCAGGTTCAC
               *..*.** *******.***..*.***..*.**********.***********.******

parental       GTGGTTCTCCTGCCTCAGCCTCCTGAGTAGCTGGGATTATAGGCCTGCATCACCATGCCT
NOT9 KO #5(WT) ACCATTCTCCTGCCTCAGCCTCCAGAGTAGCTGGGACTACAGGCGCCTGCCACCACGCCC
               ....*******************.************.**.****......*****.***.

parental       GGCTTTTTTTTTTTTTTAATTTTTAGTAGATACGGAGTTTCACCATGTTGGTCAGGCTGAT
NOT9 KO #5(WT) AGCTAATT---TTTTGTATTTTTAGTAGAGATGGGGTTTCACCGTGTTAGCCAGGATGGT
               ..***.**   ****..************.*.**.********.****.*.****.**.*

parental       CTTGAACTCCTGACCTCGTGATCCACCACCTTG-GCCTCCCAAAATGTTGAGATTACAG
NOT9 KO #5(WT) CTCGATCTGCTGACCTCGTGATCTGCCCGTCTCGGCCTCCCAAAATGTTGAGATTACAG
               **.**.**.**************..**....*. *************************
```

b

RNF219-WT, NOT9 KO #5, allele 2

```
                                guide A     PAM A
parental       AAGCCTGTGCCTACTACACTGGCACAAGTGGATAGAGAAAAGATCTATCAGTGGATCAAT
NOT9 KO #5(WT) AAGCCTGTGC--------------------------------------------------
               **********

                                                           PAM B guide B
parental       GAGCTGTCCAGTCCTGAGACTAGGGAAAAT-------XXXXXXXX---CCCACCTTGACA
NOT9 KO #5(WT) ---------------------------------------CTACTACACTGGCACTTGACA
                                                           .....*******

                   guide B
parental       GCACACCAGTCTAACAGAGTTTGCAATGCTCTGGCATTACTGCAATGTGTAGCATCACAT
NOT9 KO #5(WT) GCACACCAGTCTAACAGAGTTTGCAATGCTCTGGCATTACTGCAATGTGTAGCATCACAT
               ************************************************************
```

Supplementary Figure 8 | Genomic *NOT9* locus in HeLa knock-out cells (related to Figure 3). Sequence alignment of the targeted *NOT9* genomic locus of NOT9 KO clone #5 stably expressing 3xFS-RNF219-WT with the unmodified sequence of parental HeLa cells. A region of the *NOT9* genomic locus spanning the edited site was PCR amplified, sub-cloned and subjected to Sanger sequencing. The target sequence of the two guide RNAs is underlined. The two PAMs are highlighted in green. (**a**) *NOT9* allele 1 in KO clone #5 harbors a smaller deletion downstream of the guide RNA A target sequence in exon 2. (**b**) *NOT9* allele 2 in KO clone #5 harbors a larger deletion situated between the guide RNA A and B target sequences in exon 2 and 3. XXXXXXXX stands as placeholder for approximately 2 kb of sequence.

a

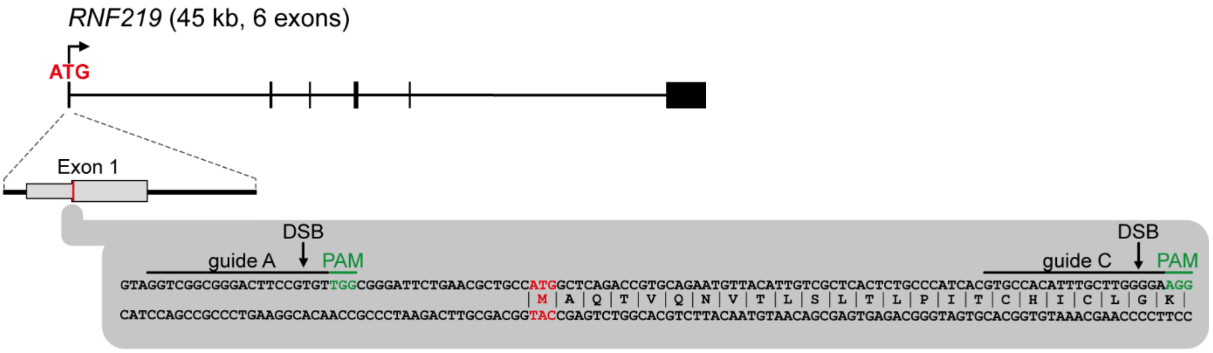

b

```
                          guide A        PAM A
parental          GCGGTAGGTCGGCGGGACTTCCGTGTTGGCGGGATTCTGAACGCTGCCAT-----------
RNF219 KO #16     GCGGTAGGTCGGCGGG---------------------------------------------
RNF219 KO #30     GCGGTAGGTCGGCGGGACTTCACCGAAAACAGTGGGCGCGCTGGCGGGTGATGATTCTGC-
                  ****************

parental          -------------------GGCTCAGACCGTGCAG---AATGTTACAT-------------
RNF219 KO #16     -------------------------------------------------------------
RNF219 KO #30     CGATATTGCTGATTCTGGTTGCGCTGGCAGTGGAGCGGGGCGTGACGTTATGGAGCGTCA-

parental          --------------------------------------TGTCGCTCACTCTGCCCATCAC-
RNF219 KO #16     -------------------------------------------------------------
RNF219 KO #30     GCGAACAGGTGGCGCAAAGCCGCACCCAGGCGGAGGAACAGTTCTTAACGTTGTTCCCGG-

                        guide C       PAM C
parental          GTGCCACATTTGCTTGGGGAAGGTAATGGGTGATTGTGGGCACAGCTACAAAACGGGGTT
RNF219 KO #16     ----------ACTTCCGGGAAGGTAATGGGTGATTGTGGGCACAGCTACAAAACGGGGTT
RNF219 KO #30     AGCAGAAGCGGATTGTGGGAAGGTAATGGGTGATTGTGGGCACAGCTACAAAACGGGGTT
                               *..*******************************************
```

Supplementary Figure 9 | Genomic *RNF219* locus in HeLa knock-out cells (related to Figure 3). (**a**) Schematic illustration of the CRISPR/Cas9 strategy by which HeLa RNF219 KO cells were generated; DSB, double-strand break; PAM, protospacer adjacent motif. (**b**) Sequence alignment of the targeted *RNF219* genomic locus of RNF219 KO clones #16 and #30 with the unmodified sequence of parental HeLa cells. A region of the *RNF219* genomic locus spanning the edited site was PCR amplified, sub-cloned and subjected to Sanger sequencing. The target sequence of the two guide RNAs is underlined. The two PAMs and the ATG start codon are highlighted in green and red, respectively. In clone #16 the sequence between the two guide RNAs was deleted, whereas clone #30 harbors a larger insertion.

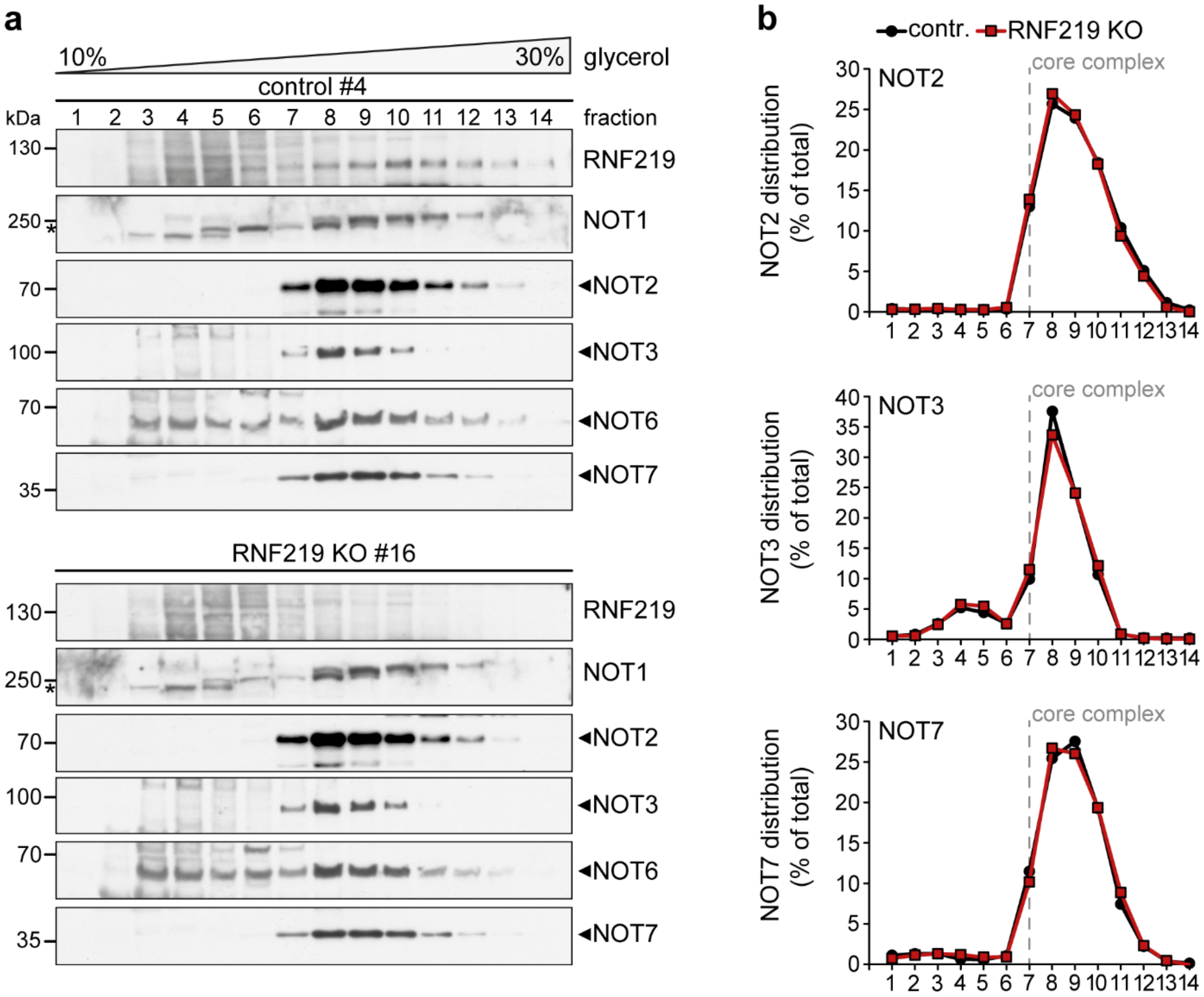

Supplementary Figure 10 | CCR4-NOT complex composition in RNF219 knock-out cells (related to Figure 3). (**a**) Co-sedimentation analysis of CCR4-NOT subunits in HeLa control clone #4 or RNF219 KO clone #16 by 10-30% linear glycerol gradient fractionation and western blotting. The core CCR4-NOT complex elutes in fraction 7-14; the asterisks denote non-specific bands. (**b**) The distribution of NOT2, NOT3 and NOT7 across the glycerol gradient was quantified from blots shown in (a); n = 1. Source data for panels **a-b** are provided as a Source Data file.

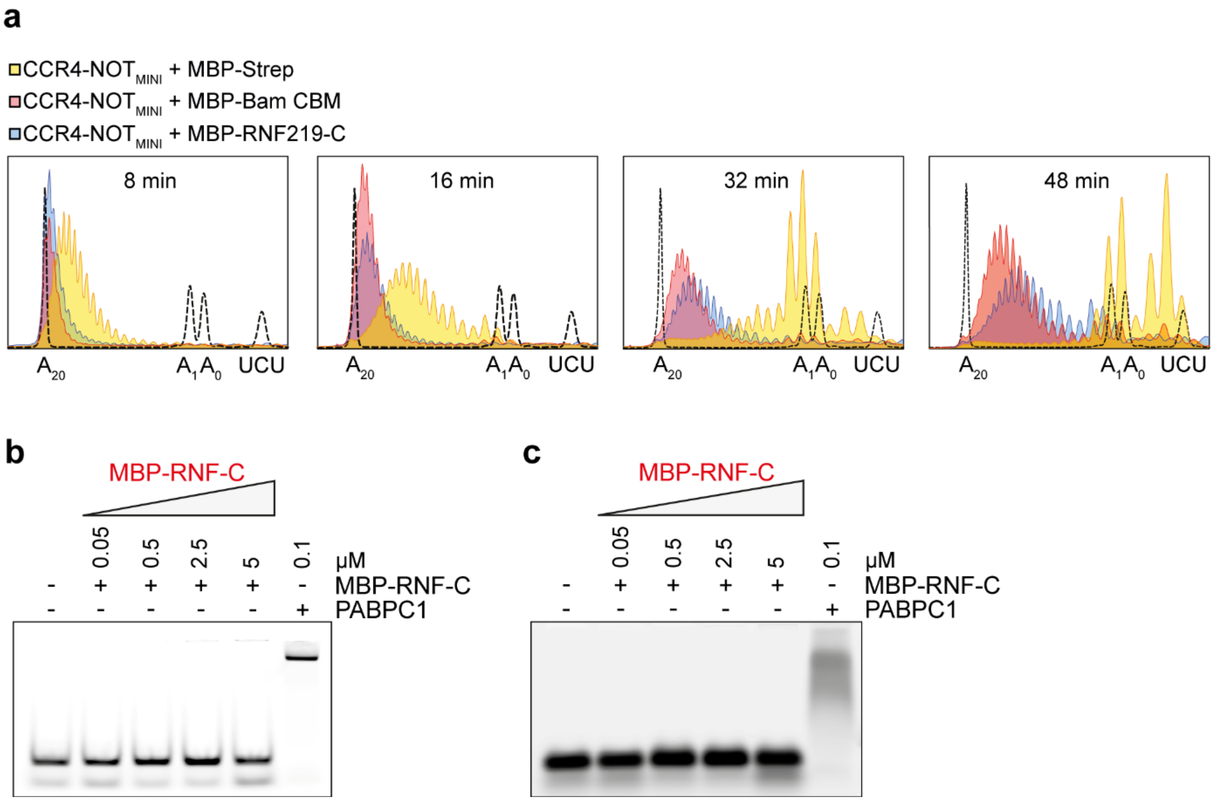

Supplementary Figure 11 | Densitometric analysis of *in vitro* deadenylation assays and *in vitro* RNA binding assays (related to Figure 4). (**a**) To quantify poly(A) tail shortening, densitometric profiles were taken at each time point of the *in vitro* deadenylation assays shown in Fig. 4b. The assay conditions are color-coded as follows: CCR4-NOT$_{MINI}$ + MBP (yellow), CCR4-NOT$_{MINI}$ + MBP-Bam CBM (red), CCR4-NOT$_{MINI}$ + MBP-RNF219-C (blue). The position of markers is indicated by black dotted lines. (**b**) EMSA with 50 nM of the 7-mer-A_{20} RNA incubated with increasing concentrations (0.05–5.0 µM) of MBP-tagged RNF219-C and, as positive control, 0.1 µM PABPC1. RNA-protein complexes were analyzed by electrophoresis on a 6% nondenaturing polyacrylamide gel. (**c**) EMSA as in (b) analyzed on a 2% TBE/agarose gel. Source data for panels **b-c** are provided as a Source Data file.

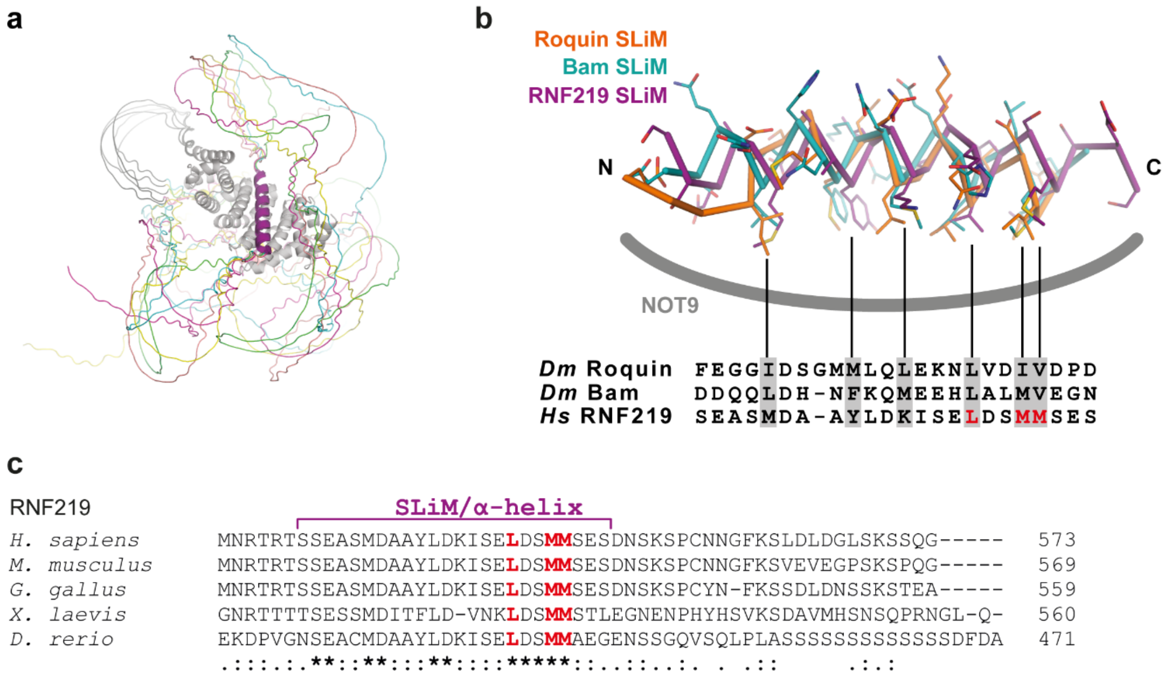

Supplementary Figure 12 | Structural and amino acid sequence conservation of the RNF219 SLiM (related to Figure 5). (**a**) Five structures predicted by AlphaFold2 of the RNF219 SLiM bound to NOT9. The models were aligned on NOT9 (gray cartoon) with polyglycine linkers shown as gray ribbons and the disordered parts of RNF219-C as individually colored ribbons. The RNF219 helical SLiMs from all five models are shown as purple cartoons. (**b**) Superposition of the Roquin, Bam and RNF219 SLiMs bound to NOT9. The backbones are shown in ribbon representation, and side chains are shown as sticks. NOT9 is indicated as a thick gray line. Sequence alignment of the SLiMs is shown below. Hydrophobic residues interacting with NOT9 are highlighted in grey. Critical residues that mediate direct interaction of the RNF219 SLiM with NOT9 are highlighted in red. (**c**) Amino acid sequence conservation analysis of the RNF219 SLiM. The alignment covers a portion of the C-terminal low-complexity region of RNF219 homologues in human (*Homo sapiens*), mouse (*Mus musculus*), chicken (*Gallus gallus*), frog (*Xenopus laevis*) and fish (*Danio rerio*). The SLiM residues which directly contact NOT9 are highlighted in red.

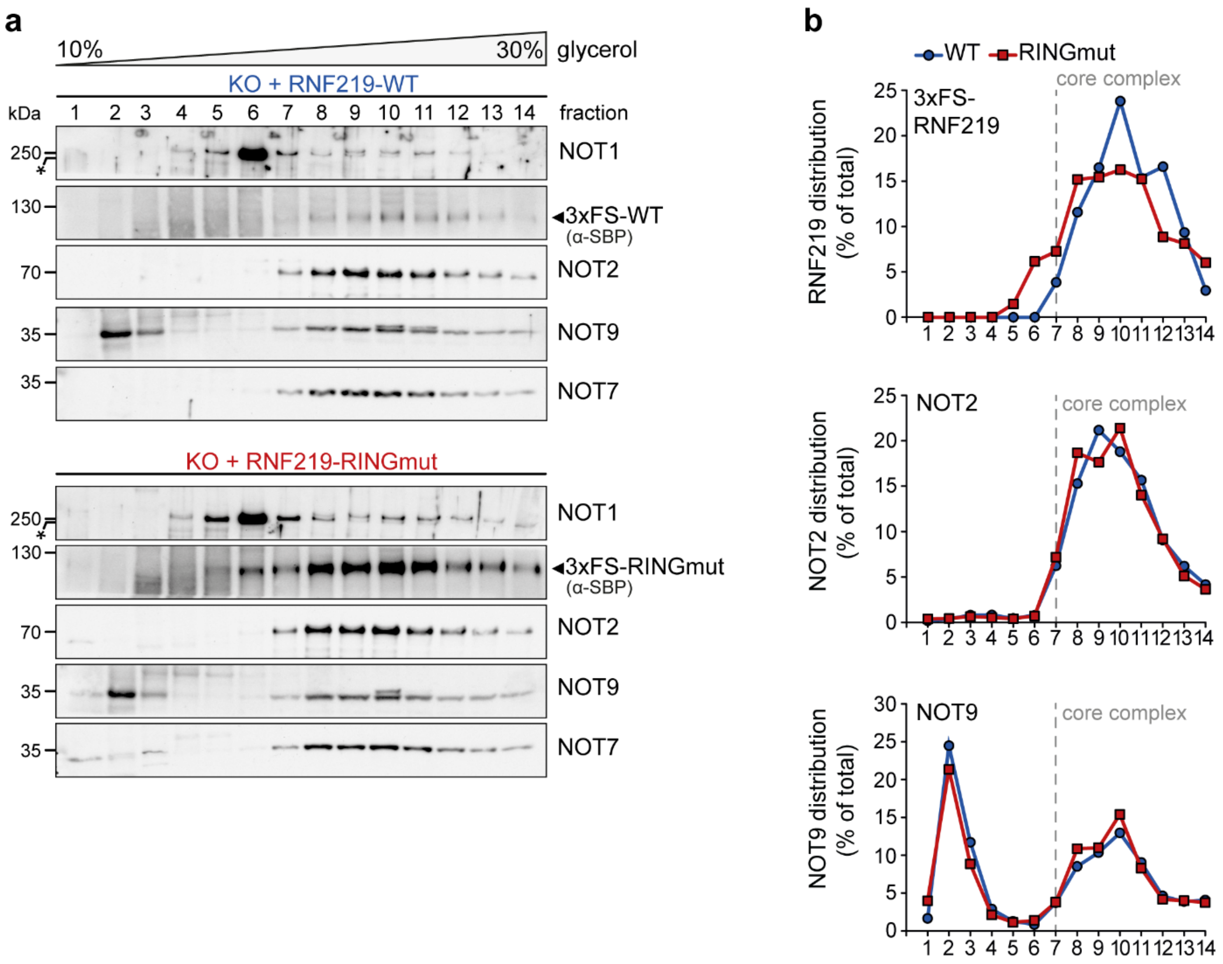

Supplementary Figure 13 | 3xFS-RNF219-WT and -RINGmut associate with the CCR4-NOT complex (related to Figure 7). (**a**) Co-sedimentation analysis of CCR4-NOT subunits in RNF219 KO cells stably expressing 3xFS-RNF219-WT or -RINGmut by 10-30% linear glycerol gradient fractionation and western blotting. The core CCR4-NOT complex elutes in fraction 7-14; the asterisks denote non-specific bands. (**b**) The distribution of 3xFS-RNF219, NOT2 and NOT9 across the glycerol gradient was quantified from blots shown in (a); n = 1. Source data for panels **a-b** are provided as a Source Data file.

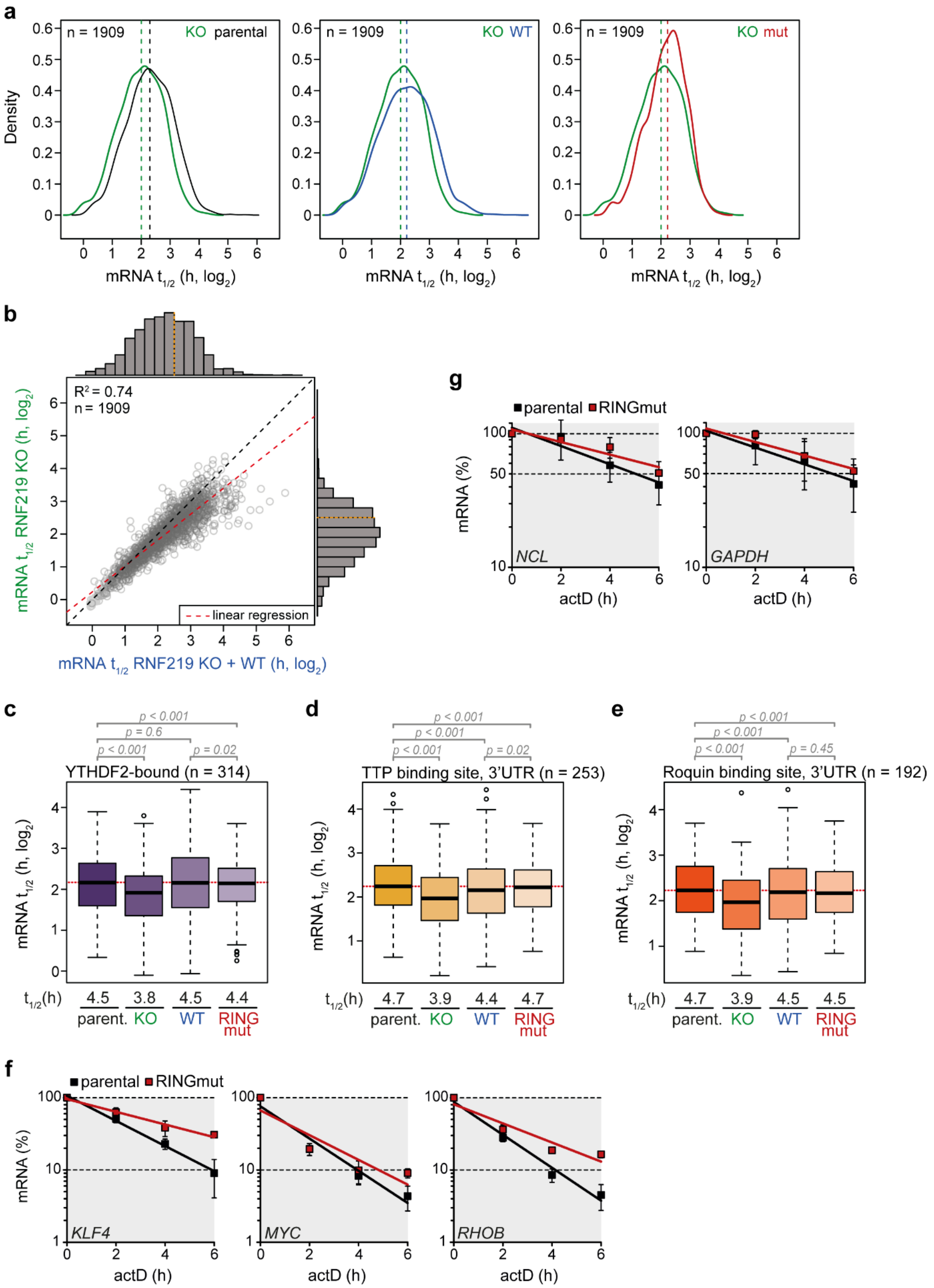

a
n = 1909
KO parental
KO WT
KO mut
Density
mRNA t1/2 (h, log2)
b
R² = 0.74
n = 1909
linear regression
mRNA t1/2 RNF219 KO (h, log2)
mRNA t1/2 RNF219 KO + WT (h, log2)
c
p < 0.001
p = 0.6
p < 0.001
p = 0.02
YTHDF2-bound (n = 314)
mRNA t1/2 (h, log2)
t1/2(h) 4.5 3.8 4.5 4.4
parent. KO WT RING mut
d
p < 0.001
p < 0.001
p < 0.001
p = 0.02
TTP binding site, 3'UTR (n = 253)
mRNA t1/2 (h, log2)
t1/2(h) 4.7 3.9 4.4 4.7
parent. KO WT RING mut
e
p < 0.001
p < 0.001
p < 0.001
p = 0.45
Roquin binding site, 3'UTR (n = 192)
mRNA t1/2 (h, log2)
t1/2(h) 4.7 3.9 4.5 4.5
parent. KO WT RING mut
f
parental RINGmut
mRNA (%)
KLF4
MYC
RHOB
actD (h)
g
parental RINGmut
mRNA (%)
NCL
GAPDH
actD (h)

Supplementary Figure 14 | Analysis of mRNA decay in HeLa RNF219 knock-out and rescue cell lines (related to Figure 7). (**a**) Density plots depicting the distribution of mRNA half-lives in parental HeLa vs. RNF219 KO cells (left), RNF219 KO cells stably expressing 3xFS-RNF219-WT vs. RNF219 KO cells (middle), and RNF219 KO cells stably expressing 3xFS-RNF219-RINGmut vs. RNF219 KO cells (right). Dashed lines indicate median mRNA half-lives. (**b**) Scatter plot and marginal histograms depicting mRNA half-lives in RNF219 KO vs. RNF219 KO cells stably expressing 3xFS-RNF219-WT, measured as in Fig. 7b. (**c**) Box-and-whisker plot depicting half-lives of YTHDF2-bound mRNAs[2], representing a subgroup of the dataset in Fig. 7c. Median mRNA half-lives are indicated below the graph; *p*-values were calculated by two-sided, paired Wilcoxon rank sum test; parent. vs. KO $p = 1.7\times10^{-46}$; parent. vs. WT $p = 0.6$; parent. vs. RINGmut $p = 3.9\times10^{-6}$; WT vs. RINGmut $p = 0.02$. (**d**) Same as in (c) for TTP/ZFP36-bound mRNAs[3]. *p*-values were calculated by two-sided, paired Wilcoxon rank sum test; parent. vs. KO $p = 5.6\times10^{-36}$; parent. vs. WT $p = 8.1\times10^{-8}$; parent. vs. RINGmut $p = 7.2\times10^{-6}$; WT vs. RINGmut $p = 0.02$. (**e**) Same as in (c) for Roquin/RC3H1-bound mRNAs[4]. *p*-values were calculated by two-sided, paired Wilcoxon rank sum test; parent. vs. KO $p = 1.7\times10^{-28}$; parent. vs. WT $p = 6.8\times10^{-6}$; parent. vs. RINGmut $p = 1.7\times10^{-8}$; WT vs. RINGmut $p = 0.45$. (**f**) Decay of IEG mRNAs *KLF4*, *MYC* and *RHOB* was measured in parental HeLa or RNF219 KO cells stably expressing 3xFS-RNF219-RINGmut. Transcription was shut-off with 5 µg/ml actinomycin D (actD). Total RNA was extracted at regular time intervals and analyzed by qRT-PCR. 18S rRNA was used for normalization; values are presented as mean ± SD (n = 3); the corresponding mRNA half-lives are shown in Fig. 7f. (**g**) Decay of normally stable *GAPDH* and *NCL* mRNA was measured in parental HeLa or RNF219 KO cells stably expressing 3xFS-RNF219-RINGmut following treatment with 20 nM RMD for 16 hours. Total RNA was prepared as described in (f) and analyzed by qRT-PCR. 18S rRNA was used for normalization; values are presented as mean ± SD (n = 5); the corresponding mRNA half-lives are shown in Fig. 7h. In all box-and-whisker plots the median is indicated as center line, the

interquartile range as box and the whiskers extend to the most extreme datapoint that is no more than 1.5 times the interquartile range from the box. Source data for panels **a-g** are provided as a Source Data file.

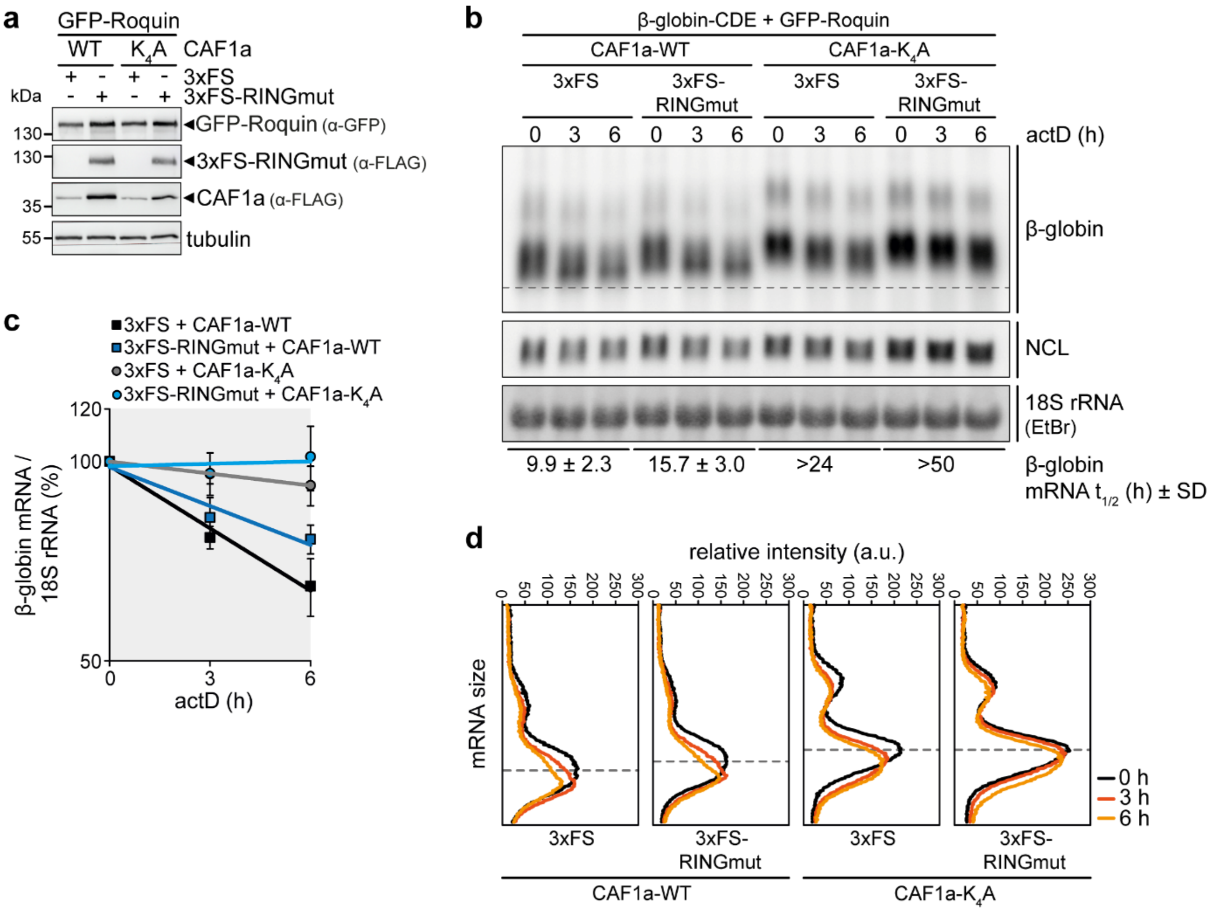

Supplementary Figure 15 | RNF219-mediated mRNA stabilization is independent of CAF1a acetylation. (**a**) Western blot analysis of HeLa cells transiently co-expressing GFP-Roquin, FLAG-SBP-tagged CAF1a-WT or -CAF1a-K4A together with 3xFS-RNF219-RINGmut or 3xFS alone. (**b**) Northern blot analysis of *β-globin* reporter mRNA stability. The CDE-containing reporter was transiently co-expressed with epitope-tagged proteins described in (a). Transcription was shut-off with 5 µg/ml actD. Total RNA was extracted at 3 h intervals and subjected to northern blot analysis using probes against *globin* and *NCL* mRNA. 18S rRNA was visualized by ethidium bromide (EtBr) staining after blotting. *β-globin* mRNA was normalized to 18S rRNA to calculate its half-life. (**c**) Quantification of *β-globin* mRNA decay measured in (b); values are presented as mean ± SD (n = 4); 18S rRNA was used for normalization. (**d**) Deadenylation was visualized by densitometric analysis of the *β-globin* mRNA signal shown in (b). Source data for panels **a-d** are provided as a Source Data file.

Supplementary References

1 Wang, Z. *et al.* Genome-wide mapping of HATs and HDACs reveals distinct functions in active and inactive genes. *Cell* **138**, 1019-1031 (2009).

2 Wang, X. *et al.* N6-methyladenosine-dependent regulation of messenger RNA stability. *Nature* **505**, 117-120 (2014).

3 Mukherjee, N. *et al.* Global target mRNA specification and regulation by the RNA-binding protein ZFP36. *Genome Biol.* **15**, R12 (2014).

4 Murakawa, Y. *et al.* RC3H1 post-transcriptionally regulates A20 mRNA and modulates the activity of the IKK/NF-kappaB pathway. *Nat Commun* **6**, 7367 (2015).
