# Supplementary material for: RNF219 attenuates global mRNA decay through inhibition of CCR4-NOT complex-mediated deadenylation
Source: Nat Commun. 2021 Dec 9;12:7175. doi: 10.1038/s41467-021-27471-6 (PMC8660800; doi:10.1038/s41467-021-27471-6)
Supplement: Supplementary file 3 — Description of Additional Supplementary Files [file 41467_2021_27471_MOESM3_ESM.pdf]

Title: Supplementary Data 1:
Description: SILAC AP-MS data derived from four independent biological replicates of a FLAG-based FST-NOT1 purification from parental or FST-NOT expressing HeLa cells (related to Figure 1).

Title: Supplementary Data 2:
Description: Transcriptome-wide mRNA half-lives measured in RNF219 KO HeLa cells or RNF219 KO cells stably expressing 3xFS-RNF219-WT or 3xFS-RNF219-RINGmut (related to Figure 7).

Title: Supplementary Data 3:
Description: Half-lives of IEGs, YTHDF2-, TTP- or Roquin-bound mRNAs measured in RNF219 KO HeLa cells or RNF219 KO cells stably expressing 3xFS-RNF219-WT or 3xFS-RNF219-RINGmut (related to Figure 7).

Title: Supplementary Data 4:
Description: Sequences of DNA and RNA oligonucleotides used in this study.
