# Supplementary material for: RNF219 attenuates global mRNA decay through inhibition of CCR4-NOT complex-mediated deadenylation
Source: Nat Commun. 2021 Dec 9;12:7175. doi: 10.1038/s41467-021-27471-6 (PMC8660800; doi:10.1038/s41467-021-27471-6)
Supplement: Supplementary file 8 — Reporting Summary [file 41467_2021_27471_MOESM8_ESM.pdf]

nature research

Corresponding author(s): Georg Stoecklin, Eugene Valkov

Last updated by author(s): 04.11.2021

Reporting Summary

Nature Research wishes to improve the reproducibility of the work that we publish. This form provides structure for consistency and transparency in reporting. For further information on Nature Research policies, see our Editorial Policies and the Editorial Policy Checklist.

Statistics

For all statistical analyses, confirm that the following items are present in the figure legend, table legend, main text, or Methods section.

n/a	Confirmed	
[ ]	[x]	The exact sample size (n) for each experimental group/condition, given as a discrete number and unit of measurement
[x]	[ ]	A statement on whether measurements were taken from distinct samples or whether the same sample was measured repeatedly
[ ]	[x]	The statistical test(s) used AND whether they are one- or two-sided *Only common tests should be described solely by name; describe more complex techniques in the Methods section.*
[x]	[ ]	A description of all covariates tested
[x]	[ ]	A description of any assumptions or corrections, such as tests of normality and adjustment for multiple comparisons
[ ]	[x]	A full description of the statistical parameters including central tendency (e.g. means) or other basic estimates (e.g. regression coefficient) AND variation (e.g. standard deviation) or associated estimates of uncertainty (e.g. confidence intervals)
[ ]	[x]	For null hypothesis testing, the test statistic (e.g. F, t, r) with confidence intervals, effect sizes, degrees of freedom and P value noted *Give P values as exact values whenever suitable.*
[x]	[ ]	For Bayesian analysis, information on the choice of priors and Markov chain Monte Carlo settings
[x]	[ ]	For hierarchical and complex designs, identification of the appropriate level for tests and full reporting of outcomes
[x]	[ ]	Estimates of effect sizes (e.g. Cohen's d, Pearson's r), indicating how they were calculated

Our web collection on statistics for biologists contains articles on many of the points above.

Software and code

Policy information about availability of computer code

Data collection	Collection and processing of mass spectrometry data: MaxQuant v1.6.12.0;<br>Detection of fluorescence of RNA gels: Typhoon Biomolecular Imager software (Cytiva);<br>Collection of western blot images: Evolution-Capt V18 software (Vilber);<br>Collection of qRT-PCR data: QuantStudio 5 software (Thermo Fisher Scientific);<br>Collection of RNA-Seq data: NextSeq System Suite v2.2.0 (Illumina)
Data analysis	Analysis of proteomics data: Perseus v1.6.2.1;<br>Quantification of blots and gels: ImageJ v1.52;<br>Analysis and processing of proteomics and RNA-Seq data: R v3.6.3;<br>DNA and amino acid sequence alignments: Clustal Omega;<br>Quantification of in vitro deadenylation experiments: ImageQuant TL v8.2;<br>Mapping of RNA-Seq reads: STAR v2.5.3a;<br>Summation of read counts on gene level: Subread package v1.6.3;<br>Data visualization and analysis: GraphPad Prism v8.4.1, Microsoft Excel 2019;<br>Calculation of transcriptome-wide mRNA half-lives with an in-house-developed R script: The pipeline used to calculate mRNA half-lives from RNA-Seq data has been deposited at the OSF (https://osf.io/vskje/?view_only=13961ac6d5cd4d3ba3521615cc38fe47);<br>Structure prediction: AlphaFold2;<br>Visualization of ChIP-Seq data: Integrative Genomics Viewer v2.9.4

For manuscripts utilizing custom algorithms or software that are central to the research but not yet described in published literature, software must be made available to editors and reviewers. We strongly encourage code deposition in a community repository (e.g. GitHub). See the Nature Research guidelines for submitting code & software for further information.

Data

Policy information about availability of data

All manuscripts must include a data availability statement. This statement should provide the following information, where applicable:

- Accession codes, unique identifiers, or web links for publicly available datasets
- A list of figures that have associated raw data
- A description of any restrictions on data availability

Raw and analyzed data of RNA-Seq experiments have been deposited at the GEO (https://www.ncbi.nlm.nih.gov/geo/) under the accession number GSE172019. Raw and processed mass spectrometry data have been deposited at the ProteomeXchange Consortium via the PRIDE partner repository with the dataset identifier PXD027237. Source data of experiments shown in Figures 1b-e, 2b-e, 3b-e, 4b-f, 6b-d, 7a-c, 7e-h and Supplementary Figures 2a-e, 3a-c, 4a-b, 4d-f, 5, 6b, 10a-b, 11b-c, 13a-b, 14a-g, 15a-d are provided with this manuscript.
ChIP-Seq data from CD4+ T-cells (Wang et al., Cell 2010) were obtained from GEO under the accession number GSE15735.

Field-specific reporting

Please select the one below that is the best fit for your research. If you are not sure, read the appropriate sections before making your selection.

- [x] Life sciences
- [ ] Behavioural & social sciences
- [ ] Ecological, evolutionary & environmental sciences

For a reference copy of the document with all sections, see nature.com/documents/nr-reporting-summary-flat.pdf

Life sciences study design

All studies must disclose on these points even when the disclosure is negative.

Sample size	We did not use statistical methods to predetermine sample size. If not otherwise indicated, all experiments were performed minimally in triplicates. Triplicate experimental design allows for the detection of outliers and is sufficient to determine statistical significance. After having performed transcriptome-wide mRNA half-life measurements in parental HeLa and RNF219 KO cells in two biological replicates, which were reproducible and led to almost identical bulk mRNA half-life, we reasoned that two replicates are sufficient.
Data exclusions	Commonly occurring contaminants, proteins only identified by a modification site or those matching the reversed part of the decoy database were excluded from proteomics data. To exclude proteins identified with low confidence, proteins were filtered for LFQ values being present in at least 3 samples across four biological repeat experiments.<br>For the analysis of transcriptome-wide mRNA half-lives, only genes with positive half-lives and R2>0.5 were considered.
Replication	All experiments were repeated independently two to six times with similar results.
Randomization	Randomization was not relevant, because the assignment of samples was determined by our treatment of the biological material and therefore is an intrinsic property of the sample.
Blinding	Blinding was not relevant, since the automized analysis of our data left no room for subconcious manipulation due to any preconceived expectations concerning its outcome.

Reporting for specific materials, systems and methods

We require information from authors about some types of materials, experimental systems and methods used in many studies. Here, indicate whether each material, system or method listed is relevant to your study. If you are not sure if a list item applies to your research, read the appropriate section before selecting a response.

Materials & experimental systems

n/a	Involved in the study	
☐	☒	Antibodies
☐	☒	Eukaryotic cell lines
☒	☐	Palaeontology and archaeology
☒	☐	Animals and other organisms
☒	☐	Human research participants
☒	☐	Clinical data
☒	☐	Dual use research of concern

Methods

n/a	Involved in the study	
☒	☐	ChIP-seq
☒	☐	Flow cytometry
☒	☐	MRI-based neuroimaging

Antibodies

Antibodies used	For western blotting: mouse monoclonal anti-FLAG clone M2 (1:1000, Sigma-Aldrich, Cat# F3165), rabbit polyclonal anti-histone H3 acetyl K27 (1:1000, Abcam, Cat# ab4729), rabbit polyclonal anti-NOT10 (1:1000, Proteintech, Cat# 15938-1-AP), rabbit monoclonal

	anti-NOT7 clone D3M6O (1:1000, Cell Signaling Technology, Cat# 86665), rabbit polyclonal anti-acetylated lysine (1:1000, Cell Signaling Technology, Cat# 9441), rabbit polyclonal anti-RNF219 (1:500, Bethyl Laboratories, Cat# A302-540), rabbit polyclonal anti-FHL2 (1:1000, Abcam, Cat# ab12327), rabbit polyclonal anti-TNKS1BP1 (1:1000, Bethyl Laboratories, Cat# A301-438), rabbit monoclonal anti-NOT2 clone D8Z8P (1:1000, Cell Signaling Technology, Cat# 34214), rabbit polyclonal anti-NOT9 (1:1000, Proteintech, Cat# 22503-1-AP), mouse monoclonal anti-SBP clone SB19-C4 (1:1000, Santa Cruz Biotechnology, Cat# sc-101595), rat monoclonal anti-tubulin clone YL1/2 (1:1000, Abcam, Cat# ab6160), rabbit monoclonal anti-NOT6 clone E1L8F (1:1000, Cell Signaling Technology, Cat# 13415), rabbit polyclonal anti-histone H3 (1:1000, Abcam, Cat# ab1791), rabbit polyclonal anti-NOT1 (1:1000, Proteintech, Cat# 14276-1-AP), rabbit polyclonal anti-CAF1a (1:1000, kindly provided by Ann-Bin Shyu, McGovern Medical School, University of Texas), rabbit monoclonal anti-NOT3 clone E1L9S (1:1000, Cell Signaling Technology, Cat# 13300), mouse monoclonal anti-ubiquitin clone P4D1 (1:1000, Cell Signaling Technology, Cat# 3936), rabbit monoclonal anti-CAPZA1 clone EPR11210 (1:1000, Abcam, Cat# ab166892), rabbit monoclonal anti-CAPZB clone EPR10236 (1:1000, Abcam, Cat# ab175212), rabbit polyclonal anti-GFP (1:1000, Abcam, Cat# ab290), mouse monoclonal anti-HDAC1 clone 10E2 (1:1000, Santa Cruz Biotechnology, Cat# sc-81598), HRP-coupled donkey anti-rabbit (1:10000, Jackson ImmunoResearch, Cat# 711-035-152), HRP-coupled donkey anti-mouse (1:10000, Jackson ImmunoResearch, Cat# 715-035-150), HRP-coupled donkey anti-rat (1:10000, Jackson ImmunoResearch, Cat# 712-035-150), AP-coupled sheep anti-Digoxigenin (1:5000, Roche, Cat# 11093274910)
Validation	Antibodies used in this study were either validated by the manufacturer, used in the literature or validated in this manuscript.<br>Anti-histone H3 acetyl K27 (Abcam, Cat# ab4729) - Manufacturer's statement on specificity (WB): "Detects a band of approximately 17 kDa." This antibody was validated in https://doi.org/10.1038/s41467-021-21893-y.<br>Anti-NOT10 (Proteintech, Cat# 15938-1-AP) - This antibody was validated in https://doi.org/10.1093/nar/gks1133. Specific enrichment of human NOT10 was detected in FST-NOT1 IP by WB in this manuscript.<br>Anti-NOT7 (Cell Signaling Technology, Cat# 86665) - Manufacturer's statement on specificity (WB): "CNOT7 (D3M6O) Rabbit mAb recognizes endogenous levels of total CNOT7 protein." Specific enrichment of human NOT7 was detected in FST-NOT1 IP by WB in this manuscript.<br>Anti-acetylated lysine (Cell Signaling Technology, Cat# 9441) - This antibody was validated in https://doi.org/10.1038/s41467-021-23904-4.<br>Anti-RNF219 (Bethyl Laboratories, Cat# A302-540) - Specific enrichment of human RNF219 was detected in FST-NOT1 IP by WB in this manuscript. Specific depletion of human RNF219 was detected in RNF219 KO cells by WB in this manuscript.<br>Anti-FHL2 (Abcam, Cat# ab12327) - Manufacturer's statement on specificity (WB): "Detects a band of approximately 32 kDa." Specific enrichment of human FHL2 was detected in FST-NOT1 IP by WB in this manuscript.<br>Anti-TNKS1BP1 (Bethyl Laboratories, Cat# A301-438) - Specific enrichment of human TNKS1BP1 was detected in FST-NOT1 IP by WB in this manuscript.<br>Anti-NOT2 (Cell Signaling Technology, Cat# 34214) - Manufacturer's statement on specificity (WB): "CNOT2 (D8Z8P) Rabbit mAb recognizes endogenous levels of total CNOT2 protein." Specific enrichment of human NOT2 was detected in FST-NOT1 IP by WB in this manuscript.<br>Anti-NOT9 (Proteintech, Cat# 22503-1-AP) - Specificity of this antibody was validated by siRNA-mediated knock-down of human NOT9 by WB in this manuscript.<br>Anti-tubulin (Abcam, Cat# ab6160) - This antibody was validated in https://doi.org/10.15252/embr.202051851 and https://doi.org/10.1016/j.molcel.2016.08.030.<br>Anti-NOT6 (Cell Signaling Technology, Cat# 13415) - Manufacturer's statement on specificity (WB): "CNOT6 (E1L8F) Rabbit mAb recognizes endogenous levels of total CNOT6 protein." Specific enrichment of human NOT6 was detected in FST-NOT1 IP by WB in this manuscript.<br>Anti-histone H3 (Abcam, Cat# ab1791) - Manufacturer's statement on specificity (WB): "Detects a band of approximately 17 kDa." This antibody was validated in https://doi.org/10.1038/s41388-021-01664-1.<br>Anti-NOT1 (Proteintech, Cat# 14276-1-AP) - This antibody was validated in https://doi.org/10.1093/nar/gks1133 and https://doi.org/10.1016/j.molcel.2016.08.030.<br>Anti-CAF1a (kindly provided by Ann-Bin Shyu, McGovern Medical School, University of Texas) - This antibody was validated in https://doi.org/10.1016/j.molcel.2016.08.030 and https://doi.org/10.1016/j.cell.2013.04.016.<br>Anti-NOT3 (Cell Signaling Technology, Cat# 13300) - Manufacturer's statement on specificity (WB): "CNOT3 (E1L9S) Rabbit mAb recognizes endogenous levels of total CNOT3 protein." Specific enrichment of human NOT3 was detected in FST-NOT1 IP by WB in this manuscript.<br>Anti-ubiquitin (Cell Signaling Technology, Cat# 3936) - Manufacturer's statement on specificity (WB): "Ubiquitin (P4D1) Mouse mAb detects ubiquitin, polyubiquitin and ubiquitinated proteins."<br>Anti-CAPZA1 (Abcam, Cat# ab166892) - Specific enrichment of human CAPZA1 was detected in FST-NOT1 IP by WB in this manuscript.<br>Anti-CAPZB (Abcam, Cat# ab175212) - This antibody was validated in https://doi.org/10.1371/journal.pone.0169260. Specific enrichment of human CAPZB was detected in FST-NOT1 IP by WB in this manuscript.<br>Anti-GFP (Abcam, Cat# ab290) - This antibody was validated in https://doi.org/10.1016/j.cell.2013.04.016.<br>Anti-HDAC1 (Santa Cruz Biotechnology, Cat# sc-81598) - This antibody was validated in https://doi.org/10.1016/j.molcel.2016.08.030.

Eukaryotic cell lines

Policy information about cell lines

Cell line source(s)	HeLa and HEK293 cells were a kind gift from Paul Anderson, Harvard Medical School.<br>S. frugiperda Sf21 insect cells were a kind gift from Imre Berger, University of Bristol.
Authentication	HeLa and HEK293 cells were authenticated via SNP profiling by Multiplexion GmbH at DKFZ.
Mycoplasma contamination	All cell lines were regularly tested for mycoplasma contamination using a PCR Mycoplasma Test Kit (AppliChem).
Commonly misidentified lines<br>(See ICLAC register)	None.
